# Quarterly Retrospect

**Published:** 1862-04

**Authors:** 


### *Quarterly Retrospect.*

*April*, 1862.

"DOUBTLESS," quoth the author of 'Hudibras,' "the pleasure is as great of being cheated as to cheat." Is not this, in fact, the true philosophy of that dallying with the pseudo-marvellous which of late has been so much in vogue? Credulity, indeed, as Dean Swift happily remarked, is a much more peaceful possession of the mind than Curiosity. It might have been supposed that, after the many exposures of collusion, imposture, and self-deception in connexion with so-called "spirit-mediums," the subject would have been pretty well tabooed by all persons who were desirous to maintain a character for common sense, or who had not recourse to it for the mere purpose of amusement—on the principle of Aristippus, who being desired to resolve a riddle, replied, that it would be absurd to resolve that which unresolved afforded so much pleasure. But no; the subject has once more crept out, and, strange to say, under the imposing shadow of the *Times*. The modern Jupiter has, alas! condescended (much after the frail fashion of his heathen namesake) to act as usher to the small fry of modern *quasi*-spirits.

In the columns of our great contemporary, on the 13th ult., promoted to the honour of larger type and leaded lines (a dignity only conceded to leading articles and those who possess the "*Open sesame*" of the mighty journal) appeared a long account of a *Sitting with Mr. Forster*, who, it appeared, as the ordinary world then learned for the first time, was "the leading" spirit-medium in the aristocratic regions of the town. "He gives sittings" (*séances* is the word), says the *Times* correspondent, "at the rate of one guinea a head, and is so fully occupied with his spiritual business that, without *an appointment on the previous day*, there is no chance of an interview. When you are lucky enough to get your hour, you certainly have your guinea's worth. Mr. Forster gives you the right of search into the ghostly bosom of all your deceased relatives and friends, and if you question them about this little debt or t'other little mortgage, they will answer you with a straightforwardness that perhaps was by no means characteristic of their earthly career."

Is Whachum a myth? whose—

"———bus'ness was to pump and wheedle,
And men with their own keys unriddle;
To make them to themselves give answers,
For which they pay the necromancers."

Is Sidrophel a delusion? Truly in this age of red books and directories, "offices of inquiry" and domestic servants, it might be imagined that a literary man would at least have been suspicious, when he found that he must make a specific appointment with a professional agent of the outer world. But with an innocent faith, worthy that shown by Hudibras himself, the *Times* correspondent visits Mr. Forster. Had he forgotten the interview between Whachum and Ralpho before Hudibras was introduced to Sidrophel?

> "He (Whachum) ask'd him whence they came, and whither
> Their business lay? Quoth Ralpho, Hither.
> Did you not lose? Quoth Ralpho, Nay.
> Quoth Whachum, Sir, I meant your way?
> Your knight,—Quoth Ralpho, is a lover,
> And pains intol'rable doth suffer;
> For lovers' hearts are not their own hearts,
> Nor lights, nor lungs, and so forth downwards.
> What time?—Quoth Ralpho, Sir, too long,
> Three years it off and on has hung.
> Quoth he, I meant what time o' th' day 'tis.
> Quoth Ralpho, Between seven and eight 'tis."
> Why then, quoth Whachum, my small art
> Tells me the Dame has a hard heart,
> Or great estate. Quoth Ralph, A jointure,
> Which makes him have so hot a mind to her."

The *Times* correspondent is indulged with the usual raps, guesses at, and legerdemain acquisition of, names on rolled scraps of paper lying on the table, "spirit-writing,'' (Mr. Forster "scribbling on the paper with preternatural rapidity, as if unable to control his movements,'') and so forth; and, finally, with the exhibition of a "spirit-hand" in the *darkened* room. The simplicity of the comments on this part of the exhibition is exquisite:—"Now you have really a right to feel a little nervous. An indistinct something rises to your view, and growing more defined is plainly a hand, with the fingers in rapid motion. About the hand there is no mistake, nor is there a doubt about its inclination to clutch a bit of paper in its vicinity." What follows is not less marvellous in its simplicity:—

"That this is a most extraordinary exhibition no one can deny who watches it fairly through. *Granted any amount of confederacy or collusion, it is still difficult to explain how the name written by the questioner on paper can be imitated in a moment by an unseen scribe under the table, and how the arm of Mr. Forster, who never quits your side, is inscribed with a word of your own choice.* Let us add that, if you write your own name and many others on scraps of paper, and, throwing them in a heap together, touch them in succession, the affirmative raps will only be given when the right paper is touched.

"At these sittings nothing is done with the view of inspiring terror. Mr. Forster is no lean, haggard seer, but a young gentleman of a frank and even jovial aspect, *remarkably gentlemanlike and urbane in his manner, and not at all indisposed to laugh and joke in the midst of his spiritual manifestations.* Now and then he appears pained and exhausted through the work of 'mediation;' but mostly, if the party consists of gentlemen only, *he smokes his cigar amid a volley of rappings, as a veteran might write his despatches with shells flying in at his windows.* Confining ourselves to the report of phenomena, we do not pretend to determine when the spirit leaves off and the flesh begins. Mr. Forster offers his patrons a very agreeable hour; his necromancy is of the most genial kind, and, if people are frightened rather than pleased, it is not his fault, but theirs. As for that strange hand, with the twiddling fingers, why should any dull, mechanical prig attempt to destroy our amusement at watching it by some dreary exposition of physical causes? The hand does no harm, and shares with hard words the property of breaking no bones. Whatever the spirits may be, they are not malignant."

Did this correspondent never see Houdin, or Robin, or the clever "prestidigitator" who has so long amused and instructed the audience at the Colosseum? Did he for a moment reflect, when he hesitated to admit the sufficiency of confederacy or collusion to explain Mr. Forster's performance, that the part which he has told us most affected him took *place in the dark*(!)? Did it never occur to him how admirably Mr. Forster's jaunty manners might be adopted to throw him off his guard? (Does Mr. Forster take his cigar alone, by the way, when he gives sittings to gentlemen only?)

Worst of all, however, the *Times*, in a leading article, piling an Ossa of absurdity upon a Pelion of absurdities, suggests that the advocates of spiritualism should "request the Royal Society, or some other body of high repute, to appoint a mixed committee of savans and lawyers,(!) in the nature of a jury, to test such experiments as may be submitted to them?"—a proposition equivalent to this—that persons who will not use their own eyes and the modicum of common sense granted to them, and will not heed the lavishly-recorded and conclusive experience of savans on this very question, should be provided with an intellectual telescope or microscope of the highest power. As well offer a person who merely wanted the simplest eyeglass for common use a one-sixteenth of an inch Rosse lens.

Since the publication of this article in the *Times*, and the recital on which it was based, abundant evidence has come to light in the pages of the *Times*, and of the *Saturday Review*, showing that in Mr. Forster's case it is the old story of imposture and self-deception. One writer says (*Times*, 17th March)—

"Like your correspondent, I sat at a most unsteady table, and joined with others in writing names on slips of paper, and throwing them, when folded up, into the centre. Now, when a little boy in a pantomime turns round at the persuasive suggestion of the clown that a balloon is in the air, or a policeman behind him, his motley deceiver takes the opportunity to ease him of any parcel he is carrying. Such another victim as this I felt myself when I discovered two or three slips of paper missing from the table, after having turned my head round for an instant to look in vain for a spirit which the Medium informed me was standing at the back of my chair. This, added to the playful manner which the Medium had of abstractedly making hay with the remainder, naturally prepared me for the presence of the spirits whose names were written in them, though I confess I was not prepared to hear that the spirits of some individuals whom I knew to be alive and well were in the room, nor did I consequently believe the stories they told as to where and how they died. Many other equally strange stories of the vagaries of spirits are related, and I have heard that the spirit of Sophia Western quite forgot her faithful Jones on one occasion, and showed, as the Medium affirmed, signs of evident interest in one of his visitors; while the spirit of poor Göthe, after rapping hard to announce his presence, was still unable to say who he was, because his summoner had pedantically written his name in German characters. Such mistakes are, however, of little moment, and the most simple reasons suffice to explain them. 'Spirits are often uncertain,' 'are sometimes bothered,' or feel the 'antagonistic influence' of some disbeliever in the room. Like the spirit invoked by the priests of Baal, they are sometimes 'talking, or on a journey, or perhaps, like ourselves at the close of a *séance*, they are fast asleep.'

"I myself did not see any 'hands' nor any 'manifestations,' but I did see the writing on the Medium's arm. As there was evidently no deception in the matter, when I went home I put a diamond ring, such as the Medium himself wore, upon the little finger of my right hand, and turning up the coat sleeve of my left arm, I carefully imitated all his motions. With the diamond slipped round my finger I gently scratched on the skin two letters, not beautifully rounded ones, but sharp and angular, like those I had seen on the Medium's own arm. After rubbing the part where I had scratched with the diamond for a few seconds, I took my hand away, and there gradually appeared upon the skin two letters—two mystic letters—which, according to different imaginations, would assume the appearance of 'characters of blood,' or of 'initials of fire.'

"You suggest in your article of Saturday last, that a committee of the Royal Society should sit upon a Medium. Alas! we hear of too many men of education who are believers in spirit-rapping; men who were better employed doubtless in their youth than in visiting the booths of conjurors at a fair, or in trying to discover under which thimble a certain pea could be concealed. Let a Medium rather be tried by a jury composed of those gipsies or fortune-tellers who are daily brought before the magistrates for obtaining money under false pretences, and let us hear what they will say upon the subject."

Another writer (*Times*, March 22nd) says:—

"The Medium's charge confines his general audience to those whose family history is recorded in the *Peerage* and similar books. His professed dealing with deceased friends draws those who suffer and are least fitted to detect imposture. Grief, with its tearful eyes, trembling awe, veneration, and blank wonder, are ill fitted to cope with an imposture. Argument is useless where men wish to be deceived, but yet the cheat is transparent. The whole system is based on the assertion that dead men's ghosts follow a medium; the most famous of the tribe assured me in an unguarded moment that if he were haunted by a spirit it would drive him mad.

"The system of communication is by raps. I saw the same man rapping, while he asserted that the spirit of my aunt was communicating with him by raps. I saw the muscular motion in one knuckle of his right hand which he masked with his left arm, and each muscular contraction answered to each sound as a pendulum answers to the tick of a clock. I tried the experiment, and produced the same sound by moving the same muscle, so as to jerk the back of the nail of the forefinger against an edge cut in the side of a pencil, the point of which was pressed hard against the table. When the medium said that my dead aunt rapped for him, and had no sympathy with me, and when he made an audible noise with a pencil, visibly before my eyes, it was a barefaced wicked attempt to deceive through human affections; but when he did that which I had done that evening at dinner, and kicked up the whole table with his knee, it was simply ludicrous, and there was a general shout of laughter.

"When he wrote with one hand under the table, and said it was a spirit who wrote, he said the thing which was not, for I can write with one hand; and generally it is too absurd to suppose that a room full of ghosts assemble to inform their relations that they know the month in which they died. Let them tell me how to make my fortune honestly, and I will try to believe. Table-turning did little harm, and promoted flirtation. Mesmerism breaks no bones. If a man when biologized thinks himself a pump and assumes the attitude, that is his look-out; but spirit-rapping is different. In the first place, it is obtaining money under false pretences; and in the second, it has driven hundreds of Americans as mad as March hares."

We would commend to the attention of the *Times* and its correspondent the following observation of Swift's—"When imagination is at cuffs with the senses, and common understanding, as well as common sense, is kicked out of doors, the first proselyte [a man] makes is himself."

It is curious that in the course of the quarter the *Times* has manifested a tendency to the marvellous in other respects than in Spirit-Rapping. Whether or not Bulwer Lytton's "Strange Story" is, in part, answerable for this, it is not for us to say.

In the *Times* for January 27th, an extraordinary case of *Spontaneous Combustion* is recited as having "just taken place," in the person of the Countess Cornelia, at Cesena (Romagna). The case is a story dating back to the year 1765, since which period it has been much used as a standard illustration by writers on the subject; among others by Sir David Brewster, in his letters on "Natural Magic." It is also referred to as the "most famous" of the cases on record, by Mr. Dickens, in the preface to "Bleak House;" but he fixes the date in 1731. Unfortunately, however, the authenticity of the case is not equal to that of the justly celebrated cases of Mr. Krook and of Jacob Faithful's mother.

The most important contribution of the *Times* to the Marvellous during the past quarter (21st Feb.), is, however, in the form of a letter, signed "Medicus," headed "*Is it Drei?*" and dated from Hull. The author had been interested in the report of the examination of a gipsy fortune-teller before Mr. Dayman, at the Wandsworth Police-court, in which reference is made to a bottle containing a "brown powder," mixed with water, supplied to a Mrs. King for the purpose of insuring her husband's death "within a month." The powder was reserved for analysis; but "Medicus" was desirous to offer a suggestion with regard to it, and he tells us that—

"Among other jealously-guarded secrets of the gipsy race is the art of preparing what they term the 'drei,' or 'dri,' a most deadly and insidious destructive agent, and for which medical science knows no antidote. Analysis detects no noxious properties whatever, and the most careful examination, microscopical or otherwise, shows it simply to consist of apparently harmless vegetable matter. The 'drei,' then, is merely a brown powder, obtained from a certain species of fungus forming the nearest connecting link between the animal and vegetable kingdoms, the powder consisting of an infinity of sporules. These fungoid sporules possess the peculiar property of being further developed only by intimate contact with living animal matter (as when swallowed, &c.); they then throw out innumerable greenish-yellow fibres about 12 or 18 inches in length. When the 'drei' is administered, usually in some warm drink, these sporules are swallowed, attach themselves to the mucous membrane, germinate, throw out millions of these silky fibres, which grow with awful rapidity, first producing symptoms of hectic fever, then cough, eventually accompanied by incessant spitting of blood, till death finally inevitably supervenes, usually in about a fortnight or three weeks' time. A case of this description came under my notice in Italy in 1860. Although the patient was attended by eminent physicians accustomed to deal with cases of slow poisoning, no suspicions of foul play were entertained till the day after the decease, when an autopsy being held, revealed the cause of death. The fibres, the growth of which had ceased with the cessation of the animal life and heat that had supported them, were already partially decomposed; had another day or two elapsed no trace would have been left of the foul deed. If the analysis of the mixture in question reveal no deleterious drug, let a dog or other animal be daily dosed, as the gipsy recommended, with 'three drops' in some warm vehicle. The result would show whether the brown powder is or is not the work of the famous and destructive 'drei.'"

We presume this is a hoax, and if so it tells a very creditable story of the imaginative powers of the author. The seeming precision of the description, with the absence of all positive information, is charming, and promises well for the author if he should be disposed to enter into the field of "sensational" romance writing. The tact with which "*the eminent physicians accustomed to deal with cases of slow poisoning*" are introduced into the story is very striking; also the delicate manner in which the whole pith of the matter is hidden under the vague term, "a *certain* species of fungus." If this species be the "*nearest* connecting link between the animal and vegetable kingdom," why let this little flaw of "a *certain*" rest in the story?

It is curious that this tale comes from Italy; so did that of the Countess Cornelia (Zangari and Bandi); so also the more recent story of the inoculation of many children with syphilis in the act of vaccination, which is now attracting so much attention from the profession.

*Apropos* of poisons, a case occurred at the York winter assizes, which most conclusively shows the need we have for stricter legislation on the sale of poisons than we now possess. Richard Buckle was tried before Mr. Justice Wightman, for having attempted to poison his wife. In the course of the trial, Mr. Henry Landon, a druggist, residing at Middlesborough, gave the following extraordinary evidence:—

"In June, the prisoner came to my shop, produced a two-drachm bottle, and asked me to fill it with laudanum. He stated it was for the purpose of rubbing his wife's gums, as she was suffering from toothache. I filled the bottle —a quarter of an ounce—and he took it away with him. A few days after, I saw him in the street, and in the course of a conversation I had with him he said he had a friend—a gamekeeper—in the country, who wished to poison a dog, and he asked me the best means of poisoning a dog. I stated to him that a few days previously I had poisoned a dog belonging to Mr. Appleton, a neighbour of mine, with strychnine. I saw him about three or four days afterwards, when he came up to me and said, 'I'll take that now.' I went into the shop, and he followed me. I took the bottle of strychnine off the shelf, and he made the remark, 'I should think that would poison a good many people.' I said there was sufficient there to poison perhaps 1000 people. *He then asked me how much was sufficient to poison a person. I said, a grain. He had a sort of globule of paper in his fingers, and he said, 'Is that a grain?' I placed two crystals on the point of a knife, and said that would be about a grain. I supplied him with from* 15 *to* 20 *grains.* I wrapped it in a piece of white paper, and over the ends I placed a printed label, 'Poison,' so that the powder could not be got at without destroying the label. He paid 6*d.* and took the poison away. A quarter of an ounce of laudanum might destroy life."

Do the chemists and druggists who contend for the legality and fitness of counter-practice add to their medical functions instructions for popularizing, as well as the means for practising, the art of poisoning?

In addition to the remarkable case of George Clark, tried for murder, before Mr. Justice Willes, at Newcastle, found guilty, and sentenced to death, he being lunatic at the time, and which we have reviewed elsewhere, two cases of trial for murder have occurred during the quarter, in which acquittals on the ground of insanity have taken place. The following are brief reports of these cases:—

Northern Circuit.—Jane Torkington was indicted for the wilful murder of Mary Elizabeth Torkington, her daughter, at Goole, in the West Riding of Yorkshire, on the 18th of September last.

Mr. Blanshard appeared for the prosecution, and Mr. Torr defended the prisoner.

It appeared that the prisoner, who was the daughter of a woman named Scholes, who resided at Goole, had been married six years, and was the mother of three children, to one of whom she was charged with administering opium on the night of the 17th of September, of which it died on the following day. She had lived very happily with her husband, and in very comfortable circumstances, at Stockport, but she had always been of a reserved and grave disposition, and, in consequence of bodily illness, had sunk into such a state of despondency and melancholy in July that her mother considered her then quite insane, and proposed, for the sake of change and diversion, that she should pay her a visit at Goole. She accordingly went there on the 15th of September, taking two of her children with her, and on the following day possessed herself of some opium, and there was no doubt from the evidence that she had given some to her little girl as well as to her little boy, the medical testimony proving that the little girl had died from the effects of opium. There was, however, no direct evidence of its being administered by the prisoner beyond its being stated by Mrs. Scholes, the mother, in the prisoner's presence, and without meeting contradiction from her, that "she had taken opium herself and given it to the two children." It was also proved that the prisoner had attempted self-destruction at her own house at Stockport by hanging herself to the bed on the 1st of June, and had only been cut down after having become insensible, and that she had then for some time been regarded as insane by her neighbours, and capable of dangerous conduct towards her children and others around her.

Under these circumstances a double line of defence was adopted on behalf of the prisoner, and it was urged, first, that there was no sufficient evidence of the poison having been administered by the prisoner; and secondly, that she was not of sound mind, and in support of this contention Mr. William Anderson, the surgeon of York Castle, was called, and proved that at the time of the prisoner's admission to the gaol, on the 21st of September, she was undoubtedly insane. Mrs. Pashley, the matron of the gaol, gave corroborative evidence; and the jury, after the reply of the learned counsel for the prosecution and the short summing up of his Lordship, immediately *Acquitted* the prisoner on the ground of insanity.—*Times*, Dec. 18.

Central Criminal Court, Jan. 8.—Old Court.—Before Mr. Baron Martin.—Mary Anne Hamilton, 37, a miserable-looking creature, who appeared to be almost overcome with grief, was charged upon an indictment, and also by the coroner's inquisition, with the wilful murder of her child, Henry Hamilton.

George Brown, a police constable of the T division, said: On Sunday night, the 15th of December, about 12 o'clock, I was on duty at Hammersmith, and the prisoner came up to me and inquired the way to Brook-green Police-station. I asked what she wanted to go to the station-house for, and she replied, "I want to give myself up for murdering one of my children." I asked her "When, and where?" and she said in Rebecca-court, Well-street, Oxford-street, at 6 o'clock that evening. I inquired how she did it, and she said by tying a piece of black braid tightly round its throat. I then asked her why she did it, and she said "For want. I could not bear to see it want for bread any longer." I said she had told a very dreadful story against herself, and inquired whether it was true, and she replied that it was too true. I asked her whether it was a boy or a girl, and she said it was a little boy, ten months old. I then took the prisoner to Brook-green Police-station, and before I did so she told me if I went to her house I should find that what she stated was true. After the prisoner was locked up I went to the place she had mentioned. It was the most miserable place I ever was in in my life. There was an old flock bed in the room, and I saw two children lying upon it, the oldest a girl, about two years old. This child was alive, but paralysed. The other child, a little boy, was dead, and I observed that a piece of wide black braid was tightly tied round its neck. There was not a morsel of any kind of food in the room, and all the bedding that was upon the bed was an old calico sheet. I took both the children to the workhouse, and then went back to the prisoner, and she asked me if the girl was alive, and I told her that she was, and she then said she could not hurt the girl because she was paralysed. The prisoner asked me what time her husband came home on the Sunday morning, and I told her at 4 o'clock. After the prisoner had been cautioned, she said again that it was too true, and she was very sorry.

Other evidence was given confirmatory of the dreadful state of misery and destitution in which the prisoner was placed at the time of the murder; and it was deposed that prior to the act, although always reserved and quiet, she had become "more strange and dejected than usual," and had also shown a disposition to wander about without cause. Verdict, *Not Guilty* on the ground of insanity.

